# Modelling multi-scale cell–tissue interaction of tissue-engineered muscle constructs

**DOI:** 10.1177/2041731418787141

**Published:** 2018-08-13

**Authors:** Ryo Torii, Rallia-Iliana Velliou, David Hodgson, Vivek Mudera

**Affiliations:** 1 Department of Mechanical Engineering, University College London, London, UK; 2 Centre for Computation, Mathematics and Physics in the Life Sciences and Experimental Biology (COMPLEX), University College London, London, UK; 3 Clinical Operational Research Unit, Department of Mathematics, University College London, London, UK; 4 Division of Surgery and Interventional Science, University College London, London, UK

**Keywords:** Cellular mechanoresponse, finite element analysis, agent-based method, engineered tissue growth prediction

## Abstract

Expectation on engineered tissue substitute continues to grow, and for an effective development of a functional tissue and to control its quality, cellular mechanoresponse plays a key role. Although the mechanoresponse – in terms of cell–tissue interaction across scales – has been understood better in recent years, there are still technical limitations to quantitatively monitor the processes involved in the development of both native and engineered tissues. Computational (in silico) studies have been utilised to complement the experimental limitations and successfully applied to the prediction of tissue growth. We here review recent activities in the area of combined experimental and computational analyses of tissue growth, especially in the tissue engineering context, and highlight the advantages of such an approach for the future of the tissue engineering, using our own case study of predicting musculoskeletal tissue engineering construct development.

## Introduction

Cellular mechanoresponse plays a significant role in determining the morphological and functional characteristics of tissue, as also investigated by Curtis and colleagues^1^ using mesenchymal stem cells, and is a key element to control quality of engineered tissues. Although the understanding of the mechanoresponse – in terms of cell–tissue interaction across scales – has been widened and deepened in recent years, there are still technical limitations to quantitatively monitor the mechanical or chemical processes involved in the development of native or engineered tissues. Mathematical or computational modelling has been introduced to fill the technical gaps and assist the investigation of the complex biomechanical systems. The aim of this article is to briefly review – as a form of commentary on a specific topic – the recent examples of computational modelling applied to address challenges in tissue engineering. We especially discuss skeletal muscle tissue engineering as a focus area of application based on our own experience to date. We also highlight how computational models could benefit future of the tissue engineering, using our own data as a case study.

### Muscular pathology and tissue engineering

The significance of tissue engineering in medicine continues to grow and a wide variety of applications can be found even looking only in the context of muscular diseases^2^ and traumatic wound healing.^3^ The main role of engineered tissues is to replace the native tissue that is damaged irreversibly and lost its function due to pathology or injury. Typical clinical examples include substantial traumatic injury, aggressive tumour ablation and/or prolonged denervation, which often require surgical reconstruction. Among those, muscle might be one of the most suitable body parts to be replaced by a tissue-engineered construct, primarily due to its accessibility and relatively straightforward structure compared to more complex organs such as the liver or the heart. In addition, for severe muscle injuries, conventional autologous transplantation or injection of ex vivo cultured muscle cells are not highly successful. The former is associated with the risk of causing problem in the donor site and hence ‘exacerbating’ the problem.^4^ The latter has an issue of poor cellular survival and/or immune rejection.^5^ To engineer a skeletal muscle tissue is therefore an approach taking the advantage of the two – a tissue substitute is grown in vitro until the tissue is stabilised with sufficient number of cells and then implanted as a neo-tissue.^6,7^ The tissue engineering approaches are continuously improved with the development of designed biomaterials and could offer solutions to many of the limitations of current therapies.^8,9^

The first successful skeletal muscle tissue engineering in publication is Vandenburgh et al.^10^ in 1988, in which contractile primary avian myotubes were cultured in collagen-coated tissue culture plates and maintained for 10 days. The use of adult cells with regenerative capability, such as canonical myogenic progenitor, remains a more common approach in skeletal muscle tissue engineering than the use of embryonic stem cells (ECs) because of the challenges involved in EC differentiation to skeletal muscle.^9^ Further continuous effort for innovation has been made to develop the ability to engineer skeletal muscle and other tissues in vitro, and tissue engineering is opening new doors in regenerative medicine. To date, the application of tissue-engineered muscle construct has advanced to prove its potential to treat volumetric muscle loss using muscle stem cells in a mouse model in vivo.^11^ Cross-disciplinary approaches integrating cellular biology, biomechanics, materials science and physiology^12,13^ are essential to formulate innovative solutions for complex musculoskeletal problems.

### Cell–tissue multiscale interaction

In both native and engineered tissues, the interaction or cross-talk between cells and the surrounding extracellular matrix plays a crucial role in the development and eventually the morphological and functional characteristics of the tissue. The medium of interaction is typically mechanical or biochemical: force, stiffness,^14,15^ porosity,^16^ distribution of oxygen,^17^ nutrients and other growth factors.^18^ The latter three have an obvious impact on cellular viability and proliferation, which are yet dependent on the characteristics of the extracellular matrix such as diffusivity, whereas the impact of the former three is less intuitive. In most of the interactions, the environment regulates the cellular behaviour such as migration and the cellular behaviour alters the environment via application of force or alteration of the extracellular matrix.

#### Cellular mechanoresponse

Various types of embryonic and adult stem cells,^19,20^ as well as other more differentiated cells such as fibloblasts^21^ and myoblasts,^22^ respond to mechanical stimuli. This mechanoresponse plays an important role in the growth and remodelling of tissue. Mode of response by a single cell differs widely including migration, orientation, proliferation and production of biochemical/signalling substances as summarised in Table 1. In the musculoskeletal context, the tension in muscle bundle acts as a guiding cue for muscle fibre development,^29^ which ultimately allows the longitudinal axis to become the principal direction of contraction controlled by motor neuron activity.^30^ This has also been confirmed in vitro using a cell-seeded three-dimensional (3D) collagen hydrogel set between two fixed ends,^13^ which demonstrated that a more coordinated force acting in the longitudinal axis of the engineered tissue caused a higher degree of isometric construct contraction, developed along the time. Likewise, the development of a collective force by fibroblasts, directed along the line of principal strain, was observed quantitatively in a 3D hydrogel using a custom-made measurement tool (culture force monitor (CFM)).^31^ This demonstrates that the contraction and alignment of the cells in reference to the deformation pattern of the host hydrogel allow interactive remodelling of the tissue construct. The knowledge of the cell–construct interaction then became a basis of an optimised method of growing aligned myotubes in 3D culture.^13^

**Table 1.** Some reported cellular mechanoresponse observed with myocyte and fibroblast. The focus here is the input and response, and detailed pathways should be found in each of the cited articles.

Cell type	Input	Response	Reference
Cardiac myocyte	Mechanical stress	Increased hypertrophy	Kaye et al.^23^
Skeletal muscle	Stretch	Increased hypertrophy	Perrone et al.^24^
Skeletal muscle	Ramp stretch Ramp and cyclic stretch	Myotube formation Increased proliferation	Cheema et al.^22^
Cardiac fibroblast	Deformation	Increased ECM synthesis	MacKenna et al.^25^
Mesenchymal fibroblast	Principal strain Tension (stress)	Alignment to direction Counteracting force generation Increased MMP-9 production	Eastwood et al.^26^
Dermal fibroblast	Tension (stress)	Increased ECM synthesis	Kessler et al.^27^
Dermal fibroblast	Tension (stress)	Elevated proliferation rate	Kuang et al.^28^

ECM: extracellular matrix; MMP-9: matrix metallopeptidase 9.

#### Characteristics of host materials and structure

The behaviour of the cells is also known to be dependent on the niche they inhabit and greatly affected by alterations in external cues.^13,32^ Substantial number of biomaterials, such as collagen, hyaluronan, hydroxyapatite and polyethylene glycol, are currently under investigation as 3D scaffolds for studying the effects of cell proliferation, migration, self-renewal and differentiation. 3D structure is considered important, better replicating the in vivo environment, in comparison to the rather traditional two-dimensional (2D) cell culture environment.^13^ While 2D cell culture environments are still widely used for better control and observation, an obvious advantage of the 3D structure is the geometrical similarity to most of the biological tissues, which allows the cells to interact – with other cells as well as the surrounding matrix – in all three directions. In terms of biomechanics, 3D structure allows non-planar deformation that facilitates mechanical stimuli differently from the 2D structures do. The difference of 2D/3D stress, in combination with different 2D/3D stress sensing mechanisms of cells, could end up in cell–tissue interactions in different time scales.^33^ In addition, the 3D cultures provide unique opportunities to control parameters that determine a rational structure in order to host cells efficiently. For example, porosity of the construct/scaffold directly affects motility of the cells especially in a construct made of a stiff material such as hydroxyapatite.^34^ Porosity or printed patterns could also be used to guide cells to form self-organised structure and better functional performance proven for various types of cells including fibroblasts and myoblasts in hydrogels.^35,36^

Another important characteristic of the host material is its stiffness. Matrix stiffness directly influences the differentiation of stem cells^37^ and closely related to the cell phenotype as well as remodelling of the ‘tissue’.^38^ In the context of cell migration within the tissue, porosity has a clear impact.^39^ The stiffness of the host material also affects the migration, that is, durotaxis, as demonstrated in fibroblasts in the collagen matrix with spatially varying stiffness.^14^ The influence in the other direction, that is, cell to tissue, is also observed in various pathways. Collagen synthesis^40^ as well as matrix metalloproteinase (MMP) production by fibroblasts is regulated by mechanical stress.^41^ These biochemical phenomena alter the stiffness of the surrounding tissue, either reinforced or degraded, which in turn alter the pattern of the local mechanical stress.

### Requirement for further development

Despite its significance, cell–tissue interaction is a challenging event to monitor especially in a quantitative manner. A common approach to monitor forces exerted by a single cell is an estimation based on microscopically monitored deformation of cell culture substrate that is made of a known material property.^42^ Although this approach is effective for 2D cell culture in which measurement of the substrate deformation is straightforward, this is much more challenging in 3D cell culture systems because of difficulty in measuring deformation in 3D as well as difficulty in associating deformation to cellular contraction force.^43^ On the other hand, the CFM^25^ allows direct and continuous monitoring of the force exerted by the cells seeded in a construct but the monitored force is a collective force by all cells in the construct.

Other important cellular-level parameters such as orientation, proliferation, migration and the resulting local cellular density are also not easy to measure non-invasively and continuously. Involvement of biochemical factors, for example, oxygen and nutrient distributions, makes this even more complex. Traditionally, observation of these parameters required fixation and staining of the construct, whereas in situ imaging or live cell imaging is becoming more common.^44,45^ and some non-invasive techniques have been developed^46^ although, due to potentially damaging impact of illumination, their temporal and spatial resolution may still be limited.^47^ Computational modelling, often called in silico modelling, offers a detailed insight into the cell–tissue interaction and a promising option for the future, with a rigorous validation against experimental data.

## Computational modelling of tissue engineering environments

Due to recent advancement of computing hardware, computational modelling techniques and the reference experimental data available, modelling of tissue deformation using finite element method (FEM) has become a common tool in biomedical engineering. Computational models allow quantification of any parameter in the virtual environment, even those which cannot be measured experimentally. For example, spatial distributions of local strain and stress are the key biomechanical factors in cellular mechanoresponse and can be calculated using FEM but cannot be acquired experimentally. Moreover, spatial and temporal resolution of such a model can be made as fine as one requires, as long as the computational environment and time allow, and hence it has the potential to investigate the phenomena between the time points in experimental data acquisition. Eventually, computational models can be used as a virtual testing environment to refine experimental conditions, such as to optimise scaffold design for osteochondral defect repair^16^ and skeletal muscle regeneration.^48^ The key to the success of such an approach is to effectively model the presence of cells in the tissue, which are in two different scales, that is, cells in tens of microns and the tissue in millimetre to centimetre scales, and implement the interaction between cellular-scale events and tissue-scale phenomena.^49^ The tissue-level phenomena are primarily deformation of the tissue or scaffold, which can be handled by traditional FEM. For cellular-level phenomena, there are two distinct approaches: modelling individual cells or modelling population density of the cells, each of which has advantages and limitations. The information in the different scales is exchanged to couple the two and achieve the simulation of the entire biological–biomechanical system. In the following sections, we summarise the contributions to date in the two modelling approaches to highlight advantage and limitation of each method.

### Modelling each individual cell in tissue – discrete cell modelling

Since cells are essentially discrete individuals residing within a tissue, it is intuitive to model each of them individually. Modelling individual cells has a long history in mathematical biology, started with cellular automaton^50^ that has been used to model self-organised pattern of tissue or organ based on individual cells following a certain set of rules. This method was designed to model the interaction of cells. For modelling a larger body of tissue or organ, such as vessel morphogenesis,^51^ a larger unit in a biological system is used as a ‘cell’. For example, in the vessel morphogenesis example, a portion of vessel approximately 1/3 to 1/5 of its diameter was modelled as a ‘cell’. This is an effective approach for modelling the behaviour of whole tissue but does not allow interaction across two physical scales different by orders of magnitude, such as tissue in centimetre scale and cell in tens of micrometre scale.

Agent-based model (ABM) is a more recent evolution in similar direction, in which cells are modelled as particles residing in a larger spatial domain representing a tissue. Here, the size of the individual cell is typically ignored because of the difference in scales but the behaviour of the cells such as migration, orientation and/or proliferation and their interaction with the surrounding environment, for example, production of a chemical substance or mechanical force, are modelled. This approach, in combination with FEM or fluid mechanical models to handle tissue-scale phenomena, has been utilised to understand a wide range of mechanobiological problems: cancer biology^52,53^ and vascular remodelling,^54,55^ cardiac scar healing^56^ and generic tissue engineering model to establish a virtual bioreactor.^49^ Although applications in muscular tissue engineering are still rather scarce,^48^ ABM has successfully captured cell–tissue interactions across scales. In addition, individual variation or uncertainty of cellular behaviour can be incorporated in ABM as in Monte Carlo simulations. Implementation of such stochastic nature in the model is more common in social science or epidemiological models^57^ in which the behaviour of the agent, that is, humans, inherently include considerable uncertainty but it has the potential to model the stochastic nature of cellular behaviour.

The limitation of ABM is potentially high computational cost. In the study of 3D myoblast cell culture system,^13^ the number of cells seeded in a 7.2 mL collagen construct was 6 million at the maximum. To model each of these requires a substantial computing power, and it would be even higher if a larger tissue with cells densely packed needs to be modelled.

### Cellular population density modelling – continuous cell modelling

Differently from the individual cell modelling approach, the cells can be modelled in terms of their distribution or density, similarly to the concentration of oxygen or growth factors. Cell density plays an important role in biological activities such as the contractile ability of engineered muscles^58^ and collective cell migration in wound healing.^59^ It has been incorporated in a mathematical framework for epidermal wound healing^60^ and other biological systems, and a similar approach has been applied in more complex scenarios of postsurgical wound healing including angiogenesis.^61^

An advantage of this approach is that a single computational method, that is, FEM, can be used to simulate the entire biological system, cellular behaviour, mass transport, tissue deformation and so on. This keeps the computational cost and complexity relatively small. Moreover, the cells do not have to be considered individually but only the distribution of them is required, and hence the computational cost is not dependent on the number of cells to be included. On the other hand, challenges arise with mathematically formulating the cellular behaviour analogous to physical phenomena such as diffusion.^61^ In addition, treatment of cellular density as a function of space assumes that the density distribution is smoothly continuous in space. Therefore, this approach may not be ideal for a biological system where random motion of each individual cell plays a significant role. For instance, random motion increases the possibility for cells to get in a close proximity, within the distance range they can interact, which has been reported to play an important role in cell sorting.^62^

### Challenges – parameter identification

As summarised in the previous two sections, the two major approaches to model tissue–cell interaction have their own strengths and limitations. One needs to carefully select a mathematical model framework most suitable for the application. A common challenge in both approaches however is to acquire model parameters from experimental observations. While substantial amount of data can be found in the literature in many biological systems, it is desirable to design experiments to obtain a set of specific model parameters for the chosen mathematical approach, as described in Coy et al.^63^ for nerve construct modelling accompanied by tailor-made experiments.

## A case study – integrating in vitro and in silico models of muscle construct

As an example of in silico modelling of tissue-engineered muscle construct, we simulated the development of a muscular construct over a week, experimented in Smith et al.^13^ where muscle-derived cells (MDCs) were seeded in a 3D collagen construct.

### Experimental procedure

**Figure 1.**
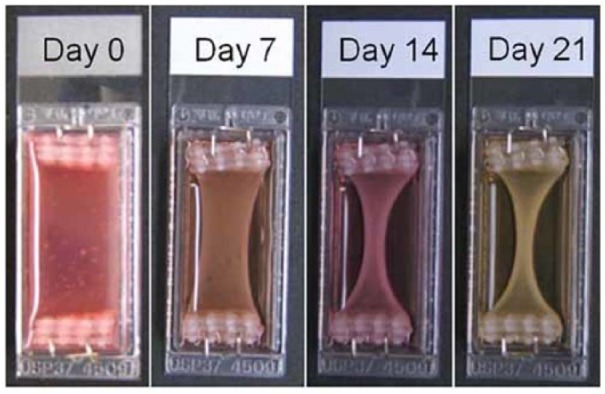
Experimental observation of the muscle construct growth. Source: Smith et al.,^13^ reproduced with permission of John Wiley and Sons.

The protocol is only briefly explained here and more details can be found in Smith et al.^13^ Primary rat MDCs were isolated from skeletal muscle samples from 1-day-old rats. The cells were suspended in growth media (GM) consisting of 20% foetal calf serum (FCS; PAA, Somerset, UK), 1% penicillin (100 U/mL) and streptomycin (100 mg/mL; P/S) in a high-glucose Dulbecco’s Modified Eagle’s Medium (DMEM) (GIBCO; Invitrogen, Carlsbad, CA, USA). The cells, counted as 5 million MDCs/cm^3^ using a hemocytometer, were seeded in an aqueous solution of type I rat-tail collagen to construct a 3D rectangular hydrogel. The collagen solution was made of 300 µL of 10× Minimum Essential Medium (MEM) solution mixed well with 2.6 mL of 2.035 mg/mL type I rat-tail collagen in 0.1M acetic acid (First Link, Birmingham, UK). The solution was neutralised with 5M NaOH drops before being mixed with the suspension of MDCs. The solution was then pipetted into a single-well chamber slide which had floatation bars at each end (Figure 1). The chamber, with the dimensions of 30 mm × 20 mm × 12 mm, was placed in a humidified incubator at 37°C for 30 min to allow collagen to set. Once set, the collagen construct was detached from the base of the mould and floated in GM at 37°C which was changed daily for a period of 14 days.

### Computational model framework

The experimental environment was modelled computationally using a combination of two modelling frameworks – FEM and ABM – to model the interaction between the cells and the construct across different scales, that is, the order of centimetre and microns.

**Figure 2.**
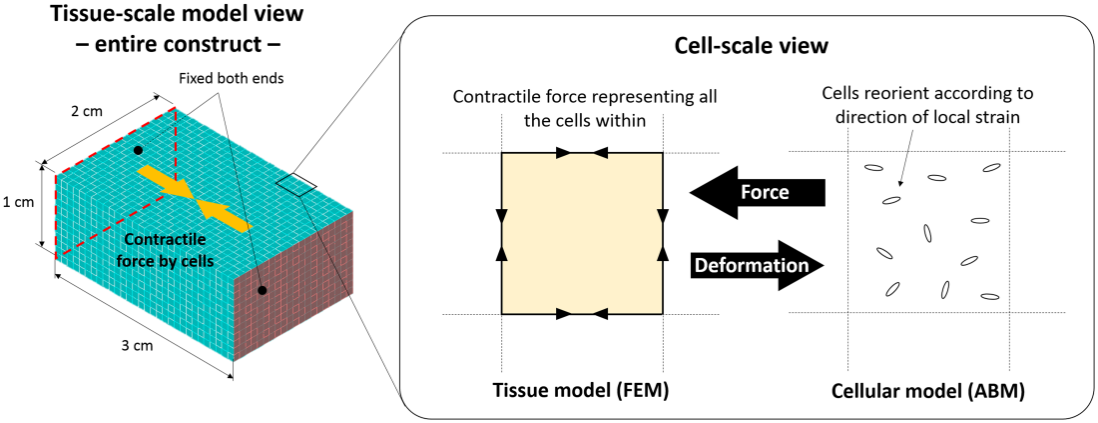
Model geometry, finite element mesh and constraint (left), and the concept of cell–construct multiscale coupling (right).

In the tissue construct scale, the construct deformation in response to the cellular contractile forces was simulated. Here, the force equilibrium equations were solved numerically using the commercial finite element (FE) package ANSYS Mechanical (ANSYS, Inc. Cannonsburg, PA, USA). Before that, the cuboidal construct was meshed into small hexahedral elements (1-mm cubes) as shown in Figure 2 and the displacement of each vertex in response to the cellular contractile forces was calculated, with the both ends of the construct fixed in space.

In the cellular scale, the behaviour of each cell was modelled as an ‘agent’ in the ABM framework implemented in the scientific computing environment MATLAB (MathWorks, Inc. Natick, MA, USA). Here, an MDC was modelled as a particle having its location (coordinates) and orientation as the associated variables. Rules were set to regulate the behaviour of each MDC and were defined as follows: (1) the cells reorient themselves in the direction of the local principal strain^29^ and (2) each cell produces a contractile force of 1.0 nN per cell. The force was estimated from a range of previous experiments to estimate the force exerted by a different number of cells, from single to millions in a collagen matrix. The studies showed a force range between 0.1 and 10 nN^25,64,65^ and we decided to take the median of the range.

The two scales are interactively coupled. Initially, the cells are randomly distributed with random orientation in the cuboidal construct:

*Step 1*. Calculate local contractile force in the direction of each cell. The local contractile force within one unit cube (element) was then calculated by integrating the forces generated by the cells within the cube.
*Step 2*. The contractile force is distributed in the edge elements such that the force within the unit cube is reflected to the FE model (Figure 2). This will allow the calculation of construct deformation and hence principal strain.
*Step 3*. In reference to the output of the FE model, cells are reoriented. Then go back to Step 1.

This iterative process was repeated every 1 h till 7 days of the computational (virtual) time. Cellular orientation, deformation of the construct, stress and strain within the construct are calculated and visualised. This iterative process is illustrated in Figure 2.

### Material properties

The construct material, that is, collagen hydrogel, was approximated as a linear elastic material with its density and normal elastic modulus being 1100 kg/m^3^ and 100 KPa,^15^ respectively. The density was determined from experimental observations and due to the fact that the gels are approximately 99% water. A Poisson’s ratio of 0.45 was chosen to model the near-incompressibility nature of the hydrogel.

### Findings from our model – potential of in silico models

**Figure 3.**
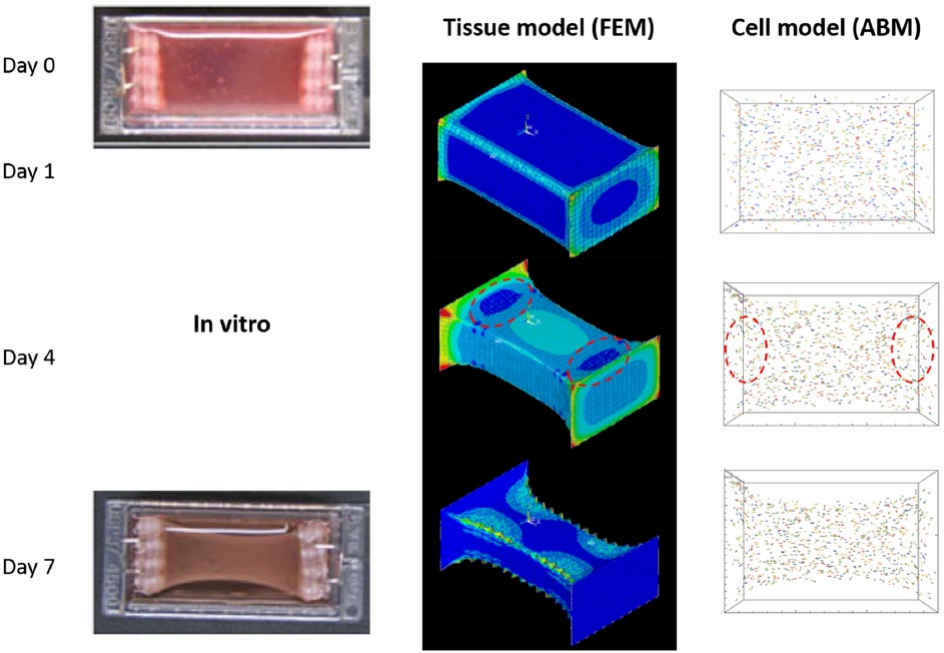
In silico model results in comparison to in vitro^13^ construct development over 7 days. The FEM results show the magnitude of principal strain (red – high and blue – low) and the ABM results show location as well as orientation of the cells by arrows. The cells are coloured randomly, to allow easy recognition of each cell. Source of in vitro images: Smith et al.,^13^ reproduced with permission of John Wiley and Sons.

Construct development over 7 days was computed and the representative results are shown in Figure 3. The tissue-scale model results show the bowing deformation of the construct, similarly to what was observed in the experiment.^13^ In the cellular scale, the randomly oriented cells at the beginning are aligned approximately in the longitudinal direction of the construct after 1 day following the line of principal strain. The long-axis alignment of the cells continues after 4 and 7 days, which corresponds to the experimental observation. The strain distribution also shows locally low strains near the fixed edges (dark blue areas in the red circle in the tissue model on day 4). These areas are known as stress-shielded area, where stress and strain are limited by the boundary fixation and the direction of the principal strain is not along the long axis of the construct. The principal strain in this area is not only low but also not in the direction of the construct’s long axis, to which the cells are not guided to align as shown in the cellular model images of Figure 3. This is another example of cellular behaviour that was observed in the experiments and successfully captured by the computational model.

**Figure 4.**
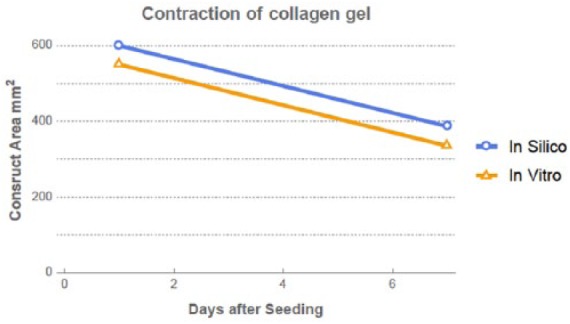
Quantitative comparison of construct development over time between in silico and in vitro models.

Figure 4 shows quantitative comparison of the construct area, looking from the top, varying over time. The in silico model successfully captured the quantitative variation of the construct area. The difference is due to the gap between the actual construct and the culturing well. The in silico model was created by taking the culturing well size, whereas the in vitro model is slightly smaller to start with. The variation of in vitro model was 39% and that of in silico model was 36%, between day 0 and day 7.

## Conclusion

Cell–tissue interaction, typically via cellular mechanoresponse, is an important factor to regulate the growth of native and engineered tissues. While more experimental methods are available and evidences have been gathered in recent years, it is still challenging to fully capture the interaction only experimentally. Integrated approaches of experimental and computational methods are therefore an effective tool for investigation in cell–tissue interaction studies. Although the number of existing examples of such a study is rather small in musculoskeletal tissue engineering, our case study shows a promising capability of an experimental data–driven computational model to represent the development of engineered tissue construct. Here, calibration of the model in reference to the experimental data, especially those from tailor-made/custom-designed experiments, is crucial to implement reality in the model. If used effectively, such an approach will fill the gap in experimentally acquired data, assist their interpretation and accelerate experimental efficiency.
